# Association Between Post-Cardiopulmonary Bypass Hemodynamics and 30-Day Mortality After Aortic Surgery: A Single-Center, Retrospective Study

**DOI:** 10.3390/jcm15207908

**Published:** 2026-10-13

**Authors:** Chang-Woo Kim, Woo-Jin Kim, Hyun-Seok Kim, Baehun Moon, Junik Park, Jeongmin Gu, Yerin Yoon, Woo-Young Seo, Sung-Hoon Kim

**Affiliations:** 1 Department of Anesthesiology and Pain Medicine, Asan Medical Center, Brain Korea 21 Project, University of Ulsan College of Medicine, Seoul 05505, Republic of Korea; kcw.amc@gmail.com (C.-W.K.); wj888.kim@gmail.com (W.-J.K.); mabn2232@gmail.com (B.M.); pjik0078@gmail.com (J.P.);; 2 Big Data Research Center, Asan Institute for Life Science, Asan Medical Center, Seoul 05505, Republic of Korea; khs0330kr@gmail.com

**Keywords:** aortic diseases, cardiopulmonary bypass, mortality, hemodynamics, blood pressure, risk assessment

## Abstract

**Background:** We evaluated phase-specific associations between perioperative hemodynamic variables and 30-day mortality following aortic surgery requiring cardiopulmonary bypass (CPB) and to assess the utility of post-CPB variables in risk stratification. **Methods:** We analyzed 691 patients who underwent aortic surgery requiring CPB. Hemodynamic variables were assessed during the preoperative, pre-incision, pre-CPB, and post-CPB phases and included heart rate (HR), systolic blood pressure (SBP), mean blood pressure (MBP), diastolic blood pressure (DBP), shock index (SI; HR/SBP), and modified shock index (MSI; HR/MBP). The primary outcome was 30-day mortality. Holm correction was applied to the between-group comparisons of hemodynamic variables. Adjusted Cox models used ridge penalization and included surgery group, CPB time, and mean post-CPB vasoactive-inotropic score. **Results:** Thirty patients (4.3%) died within 30 days. After Holm correction, significant differences between survivors and non-survivors remained only for post-CPB SBP, MBP, SI, and MSI. In adjusted Cox models, 30-day mortality was associated with higher post-CPB SI (hazard ratio [95% CI], 1.38 [1.17 to 1.64] per 0.1-unit increase; *p* < 0.001) and MSI (1.29 [1.12 to 1.47] per 0.1-unit increase; *p* < 0.001), as well as lower SBP (1.60 [1.24 to 2.05] per 10-mmHg decrease; *p* < 0.001) and MBP (2.00 [1.32 to 3.03] per 10-mmHg decrease; *p* = 0.001); in contrast, HR and DBP did not show a significant association with 30-day mortality. Kaplan–Meier analyses confirmed poorer survival across ROC-derived post-CPB thresholds for HR, SBP, MBP, SI, and MSI (all log-rank *p* ≤ 0.013). **Conclusions:** Post-CPB SBP, MBP, SI, and MSI showed significant adjusted associations with 30-day mortality and may assist in early postoperative risk stratification. These exploratory findings require external validation, and the identified thresholds should not be interpreted as treatment targets.

## 1. Introduction

Thoracic aortic surgery requiring cardiopulmonary bypass (CPB) is among the most complex surgical procedures and remains associated with substantial early postoperative risk despite advances in perioperative management and operative techniques [1,2]. Contemporary society guidelines emphasize the heterogeneity of aortic disease and the complexity of operative decision-making, underscoring the clinical importance of early postoperative outcomes in this population [3,4]. Observational studies of total aortic arch replacement similarly demonstrate significant early mortality and major morbidity, reflecting the high-acuity perioperative course typical of complex aortic operations [5,6].

Hemodynamic physiology during aortic surgery with CPB varies throughout the perioperative course [7]. Early intraoperative values are influenced by anesthetic induction, airway instrumentation, and surgical stimulation, whereas post-CPB values reflect the combined effects of ventricular performance, volume status, vasomotor tone, and separation from CPB [8,9,10,11,12,13]. Consequently, the interpretation of hemodynamic deviations may depend on the phase in which they are observed.

Modern anesthesia information systems automatically capture and archive intraoperative physiological data, enabling systematic, phase-specific evaluation of routinely available measures such as heart rate (HR) and arterial pressure [14]. Prior perioperative studies have shown that intraoperative hemodynamic patterns can be associated with adverse outcomes, including mortality and major complications. In a large prospective international cohort of patients undergoing noncardiac surgery (VISION), intraoperative HR and systolic blood pressure (SBP) were associated with myocardial injury after noncardiac surgery and subsequent clinical outcomes [15]. In cardiac surgery, intraoperative hemodynamic abnormalities have been associated with mortality, stroke, and perioperative myocardial infarction [16,17,18,19]. However, in complex aortic surgery, the prognostic value of these measures during discrete perioperative periods remains incompletely defined, and clinically interpretable thresholds have yet to be well established.

Accordingly, we evaluated perioperative hemodynamics in adults undergoing aortic surgery with CPB, focusing on the preoperative, pre-incision, pre-CPB, and post-CPB phases. We examined HR, SBP, mean blood pressure (MBP), diastolic blood pressure (DBP), shock index (SI), and modified shock index (MSI) across perioperative phases and evaluated the associations of post-CPB measurements with all-cause 30-day mortality. We also explored candidate cutoff values for parameters showing discriminatory performance using receiver-operating characteristic (ROC) curve–based methods.

## 2. Materials and Methods

### 2.1. Study Design and Population

This retrospective observational study was approved by the Institutional Review Board, which waived the requirement for written informed consent due to the study’s retrospective design. The study was conducted in accordance with relevant institutional guidelines and the Declaration of Helsinki (2013 version) [20].

We retrospectively identified adult patients (aged 18 years or older) who underwent aortic surgery with CPB between March 2019 and February 2023. Aortic surgery included procedures involving the ascending aorta, aortic arch, and descending aorta. Surgical procedures were classified into two groups according to the anatomical extent of aortic surgery: procedures limited to the aortic root and/or ascending aorta, and procedures involving the aortic arch and/or distal aorta. Procedures involving both proximal and arch or distal aortic segments were assigned to the latter group. We excluded cardiac procedures performed without CPB, CPB cases without aortic surgery, repeat operations beyond the first eligible procedure for each patient, and cases lacking complete intraoperative recordings of SBP, MBP, DBP, and HR. No data imputation was performed. Because this retrospective study included all eligible patients during the study period, no formal sample size calculation was performed.

### 2.2. Anesthesia and Perfusion Management

No uniform anesthesia or perfusion management protocol was applied to all patients during the study period. Anesthetic agents, vasoactive support, and cardiopulmonary bypass management were selected and adjusted by the treating anesthesiologist, perfusionist, and surgical team according to each patient’s clinical condition and operative requirements.

### 2.3. Data Collection and Perioperative Period Definitions

Data were obtained from the electronic medical record and the anesthesia information system. Extracted variables included demographic, clinical, and perioperative data. Preoperative heart rate and blood pressure values were derived from ward records during the 24 h period before surgery. For each patient, all available measurements recorded within this period were averaged to obtain the preoperative mean heart rate, systolic blood pressure, mean blood pressure, and diastolic blood pressure. Intraoperative HR and arterial blood pressure (ABP) values were obtained from the electronic anesthesia record, in which physiologic measurements were automatically recorded at 5 min intervals. SBP, MBP, and DBP were obtained from the recorded ABP data. For each intraoperative period, all available 5 min measurements were averaged to obtain phase-specific mean HR, SBP, MBP, and DBP values. The shock index (SI) and modified shock index (MSI) were not directly measured by the monitoring equipment but were calculated by the investigators from the extracted hemodynamic data. For each phase, SI was calculated as the phase-specific mean HR divided by the phase-specific mean SBP (SI = mean HR/mean SBP), and MSI was calculated as the phase-specific mean HR divided by the phase-specific mean MBP (MSI = mean HR/mean MBP) [21,22]. The specific manufacturer and model of the physiologic monitoring equipment were not available in the retrospectively extracted electronic medical record dataset.

The perioperative period was divided into preoperative and intraoperative periods. The intraoperative period was further subdivided into pre-incision, pre-CPB, CPB, and post-CPB phases. The pre-incision phase was defined as the interval from anesthesia induction to skin incision, the pre-CPB phase as the interval from skin incision to initiation of CPB, the CPB phase as the duration of extracorporeal circulation, and the post-CPB phase as the interval from CPB termination to the end of anesthesia. The pre-incision phase included anesthesia induction, tracheal intubation, and subsequent stabilization before skin incision and was analyzed as a single phase. The CPB phase was excluded from hemodynamic analyses because values during CPB primarily reflect extracorporeal circulation rather than native cardiovascular function. For descriptive analyses of medication exposure, vasopressor, inotrope, and antihypertensive use within each perioperative period were classified as any use versus no use. Post-CPB vasoactive support was quantified using the vasoactive-inotropic score (VIS). VIS was calculated as dopamine dose + dobutamine dose + 100 × epinephrine dose + 100 × norepinephrine dose + 10 × milrinone dose + 10,000 × vasopressin dose [23]. Doses were expressed in μg/kg/min, except for vasopressin, which was expressed in units/kg/min. Mean and maximum VIS were summarized for the post-CPB phase, and mean VIS was included in the adjusted Cox models.

### 2.4. Quantification of Post-CPB Hypotension Burden

To account for the depth and duration of post-CPB hypotension, we additionally quantified MBP burden below 65 mmHg during the post-CPB period, defined as the interval after the CPB termination to the end of anesthesia. MBP values were analyzed as a time series using linear interpolation between consecutive measurements; intervals exceeding 10 min were not interpolated. The area under the MBP threshold (AUC) was calculated as the integrated deficit below 65 mmHg (mmHg·min). The time-weighted average (TWA) burden was calculated by dividing AUC by the total post-CPB observation time. We also calculated the percentage of the post-CPB observation period during which MBP was <65 mmHg. A sensitivity analysis was performed after restricting the cohort to patients with at least 80% MBP coverage of the post-CPB observation period.

### 2.5. Statistical Analysis

Continuous variables were presented as mean ± standard deviation (SD) or median [Q1, Q3], as appropriate, and categorical variables as number (%). Normality was assessed using the Shapiro–Wilk test. Between-group comparisons were performed using Student’s *t*-test or Welch’s *t*-test for normally distributed continuous variables, the Mann–Whitney U test for non-normally distributed continuous variables, and the chi-square test or Fisher’s exact test for categorical variables, as appropriate. All tests were two-sided, and a *p*-value < 0.05 was considered statistically significant. To account for multiple comparisons of hemodynamic variables (6 hemodynamic variables × 4 perioperative phases = 24 comparisons), the *p*-values were adjusted using the Holm step-down method to control the family-wise error rate. Both unadjusted and Holm-adjusted *p*-values are reported, and statistical significance for these comparisons was determined using the Holm-adjusted *p*-values (Holm-adjusted *p* < 0.05).

The primary outcome was all-cause mortality within 30 days after surgery. Associations between post-CPB hemodynamic variables and 30-day mortality were evaluated using Cox proportional hazards regression. Time-to-event was calculated in days from the date of surgery to the date of death. Death within 30 days after surgery was considered as an event, while patients who survived beyond 30 days were censored at 30 days. The primary exposures were post-CPB mean HR, SBP, MBP, DBP, SI, and MSI. Separate unadjusted and adjusted models were fitted for each variable. Adjusted models accounted for Surgery group, CPB time and Mean VIS. Multivariable associations were estimated using Ridge-penalized Cox proportional hazards regression with an L2 penalty (penalizer = 0.01). The ridge penalty of 0.01 was selected from candidate values ranging from 0 to 0.5 using repeated five-fold cross-validation. Ridge penalization was used to reduce the risk of overfitting given the limited number of deaths. The adjustment variables represented surgical extent, CPB duration, and the intensity of vasoactive support. HR was modeled per 10-beats/min increase, SBP, MBP, and DBP per 10-mmHg decrease, and SI and MSI per 0.1-unit increase. Results are reported as hazard ratios with 95% CIs. Associations of procedure time and CPB time with 30-day mortality were additionally examined using univariable Cox regression, with both variables modeled per 10 min increase.

The discriminatory performance of post-CPB hemodynamic variables for predicting 30-day mortality was evaluated using receiver-operating characteristic (ROC) analysis and the area under the curve (AUROC). Candidate cutoff values were determined using Youden’s J statistic and rounded to clinically interpretable thresholds for Kaplan–Meier analyses. [24] Survival curves were estimated using the Kaplan–Meier method and compared using the log-rank test.

Potential nonlinear associations between continuous post-CPB hemodynamic variables and 30-day mortality were examined using restricted cubic spline Cox models with three knots placed at the 10th, 50th, and 90th percentiles of the exposure distribution. Multicollinearity was assessed using variance inflation factors, and the proportional hazards assumption was evaluated using scaled Schoenfeld residuals.

Analyses were performed using Python (v3.11.14; Python Software Foundation, Beaverton, OR, USA) with the following open-source packages: SciPy (v1.17.1; SciPy developers; https://scipy.org), statsmodels (v0.14.6; statsmodels developers; https://www.statsmodels.org), scikit-learn (v1.8.0; scikit-learn developers; https://scikit-learn.org), and lifelines (v0.30.1; Cameron Davidson-Pilon and contributors; https://lifelines.readthedocs.io).

## 3. Results

### 3.1. Study Population and Baseline Characteristics

**Figure 1:**
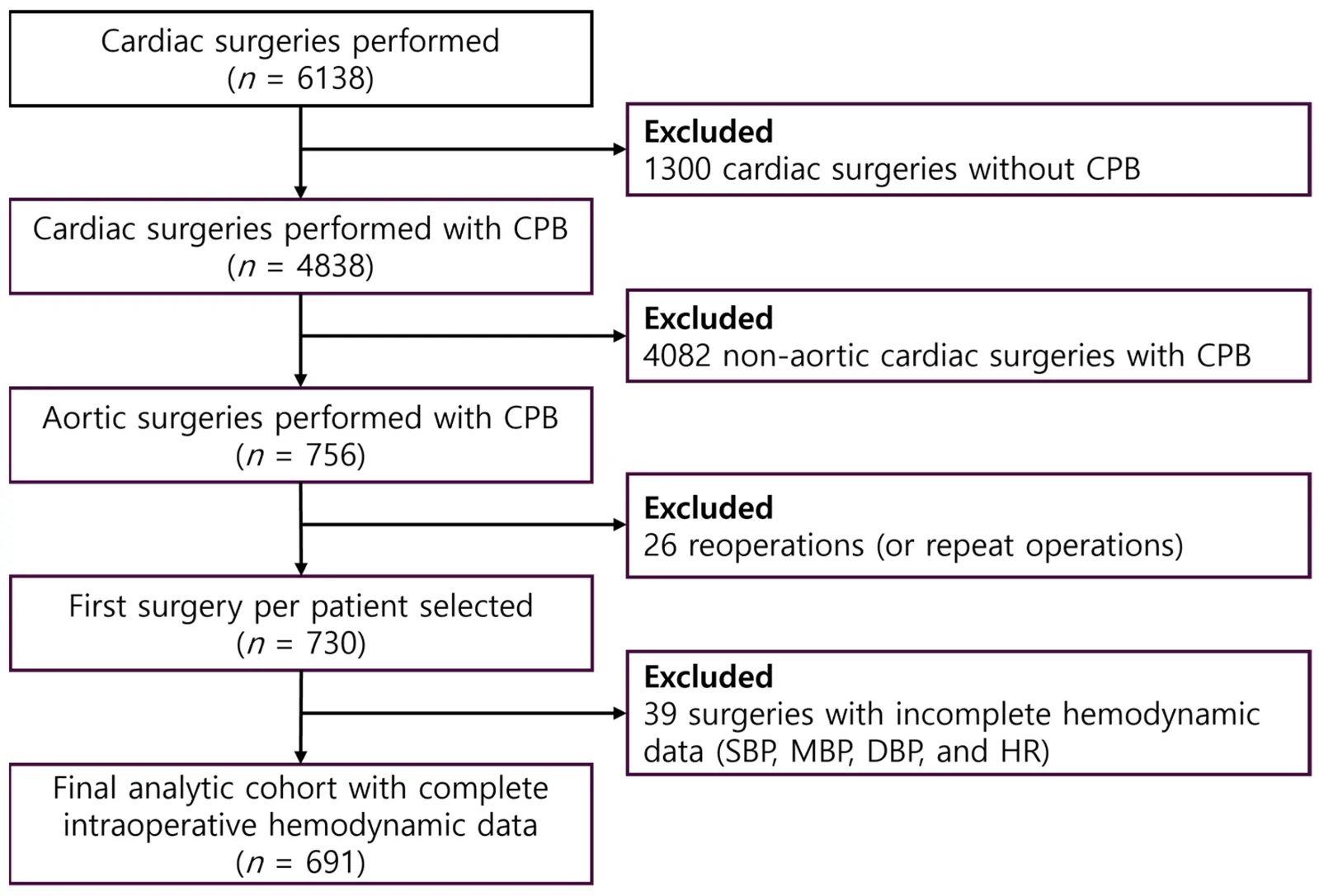
Flow diagram of patient selection. Among 6138 adult patients (18 years or older) who underwent cardiac surgery between 2019 and 2023, exclusions were applied sequentially as follows: cardiac surgeries without cardiopulmonary bypass (CPB; *n* = 1300), CPB without aortic surgery (*n* = 4082), repeat operations beyond the first eligible procedure (*n* = 26), and cases lacking complete intraoperative recordings of systolic, mean, and diastolic blood pressures and heart rate (*n* = 39). The final analytic cohort included 691 patients.

Among 6138 cardiac surgery procedures screened, 691 patients were included in the final analytic cohort, of whom 30 (4.3%) died within 30 days after surgery (Figure 1). Compared with survivors, non-survivors were older and had lower height and body weight. They were also more likely to have an ASA physical status greater than III and to have undergone emergency surgery. Procedure time and CPB time were longer in non-survivors than in survivors, while smaller differences were observed for sex, body mass index, diabetes mellitus, and hypertension (Table 1). Post-CPB mean and maximum VIS were higher in non-survivors than in survivors (both *p* < 0.001; Table 1).

### 3.2. Perioperative Hemodynamics and Medication Use According to 30-Day Mortality

Perioperative hemodynamic parameters and medication use are summarized in Table 2 and Table S1, respectively. Preoperative hemodynamic variables did not differ significantly between survivors and non-survivors. After Holm adjustment, post-CPB SBP, MBP, SI, and MSI remained significantly different (Holm-adjusted *p* < 0.05), whereas pre-incision MSI (unadjusted *p* = 0.026; Holm-adjusted *p* = 0.487) and pre-CPB HR (unadjusted *p* = 0.027; Holm-adjusted *p* = 0.495) did not. In the unadjusted medication-use comparisons, non-survivors exhibited more frequent use of dobutamine and antihypertensive medications during the pre-incision phase and more frequent dobutamine use during the pre-CPB phase. The most pronounced differences emerged during the post-CPB phase. Compared with survivors, non-survivors had significantly higher SI and MSI and lower SBP and MBP after Holm correction, whereas differences in HR and DBP were not significant after correction. Overall vasopressor and antihypertensive use after CPB did not differ significantly between groups; however, dobutamine, epinephrine, and vasopressin were used more frequently in non-survivors (Table S1). Both procedure time and CPB time were significantly associated with 30-day mortality in univariable Cox regression (Hazard ratio per 10 min increase, 1.06 [95% CI, 1.04–1.07] and 1.08 [95% CI, 1.05–1.11], respectively; both *p* < 0.001).

### 3.3. Association of Post-CPB Hemodynamic Variables with 30-Day Mortality

Given that the most consistent between-group differences were observed in the post-CPB phase, subsequent prognostic analyses focused on post-CPB hemodynamic variables (Table 3; Table S2). In ridge-penalized multivariable Cox regression models adjusted for Surgery group, CPB time, and Mean VIS, lower SBP and MBP were independently associated with increased 30-day mortality (Table 3). Notably, each 10-mmHg decrease in SBP and MBP was associated with 60% and 100% higher mortality hazards, respectively. In contrast, post-CPB HR and DBP were not independently associated with mortality after adjustment. Composite indices also remained significant predictors, with each 0.1-unit increase in SI and MSI associated with 38% and 29% higher mortality hazards, respectively. Among the covariates, CPB time remained independently associated with mortality in all models, and Surgery group was significantly associated with mortality in four of the six adjusted models, whereas Mean VIS showed a borderline association (Table S2).

### 3.4. Discrimination, Candidate Thresholds, and Survival Stratification

**Figure 2:**
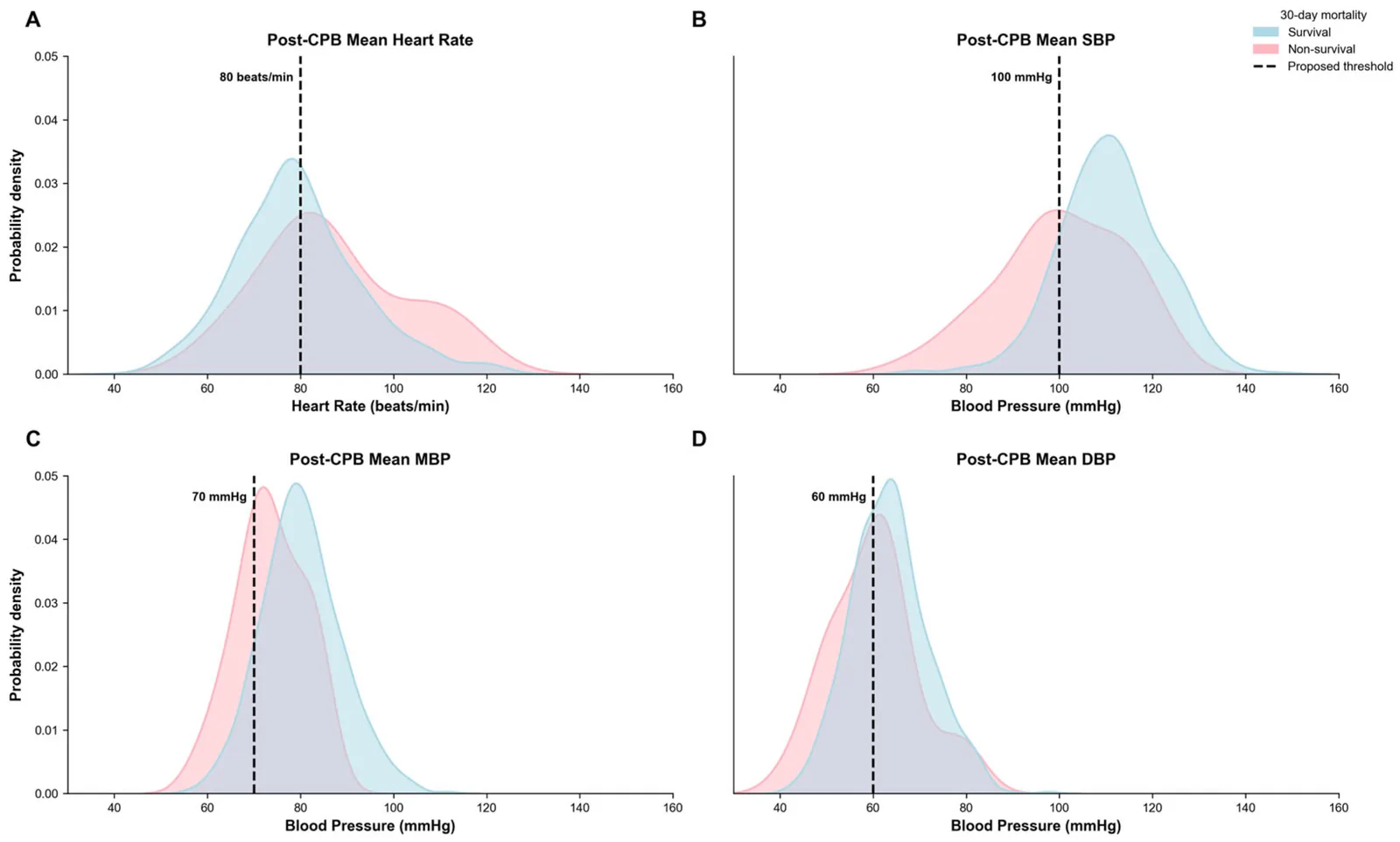
Distribution of post-CPB hemodynamic variables according to all-cause 30-day mortality. Kernel density plots comparing survivors and non-survivors for post-CPB mean (**A**) heart rate, (**B**) systolic blood pressure, (**C**) mean blood pressure, and (**D**) diastolic blood pressure. Non-survivors showed a rightward shift in heart rate and leftward shifts in systolic and mean blood pressures, whereas diastolic blood pressure distributions overlapped substantially between groups. Vertical dashed lines indicate the proposed thresholds used in the categorical analyses: 80 beats/min for heart rate, 100 mmHg for systolic blood pressure, 70 mmHg for mean blood pressure, and 60 mmHg for diastolic blood pressure.

**Figure 3:**
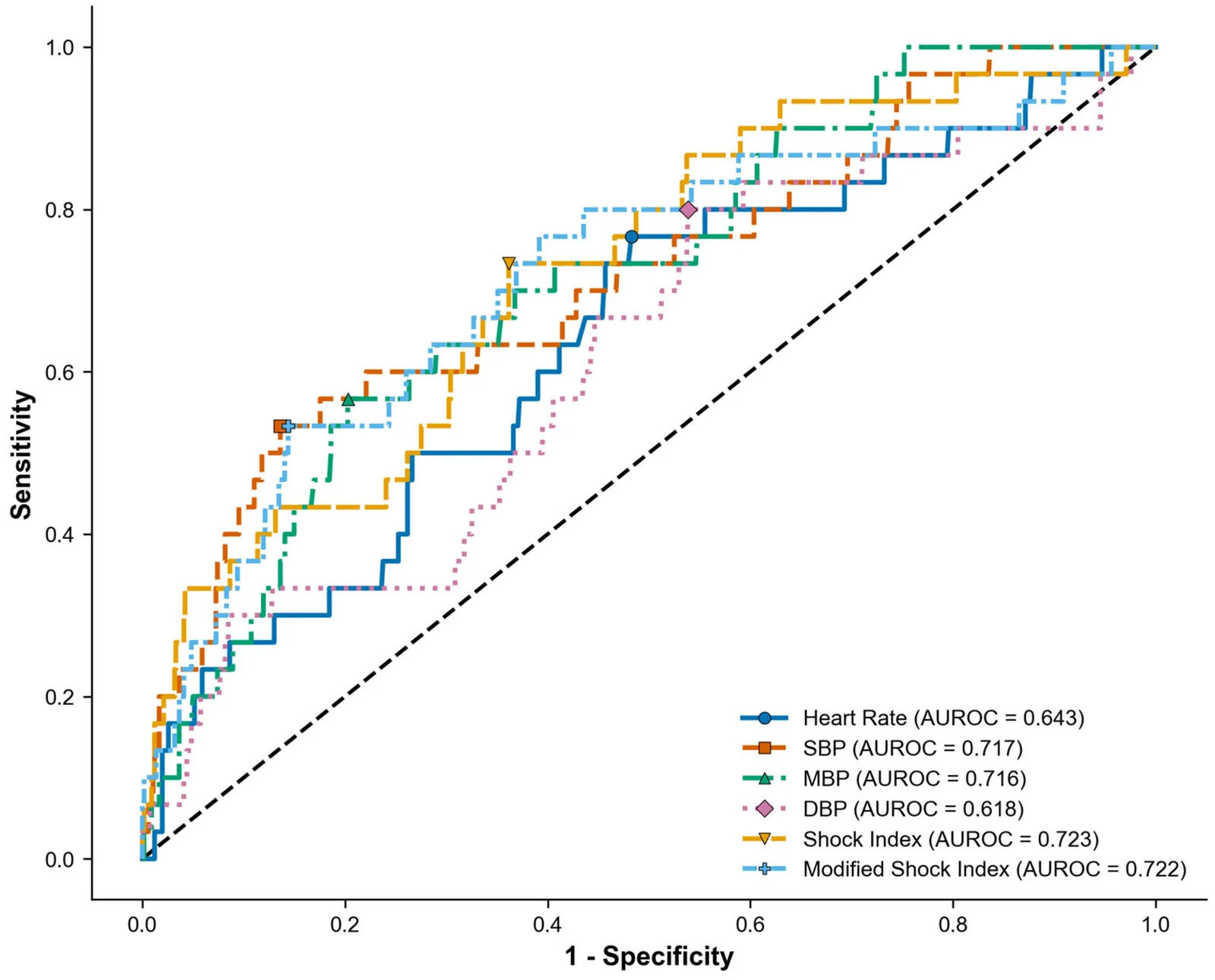
Receiver-operating characteristic curves of post-CPB hemodynamic variables for predicting all-cause 30-day mortality. Receiver-operating characteristic (ROC) curves are shown for post-CPB heart rate, systolic blood pressure, mean blood pressure, diastolic blood pressure, shock index, and modified shock index. The area under the ROC curve values are shown in the figure. The black dashed diagonal line represents no discrimination (AUROC = 0.5). Rounded thresholds used for subsequent analyses were 80 beats/min, 100 mmHg, 70 mmHg, 60 mmHg, 0.7, and 1.2, respectively.

In distribution analyses, non-survivors showed a rightward shift in HR and leftward shifts in SBP and MBP, whereas DBP distributions overlapped substantially between groups (Figure 2). ROC analysis demonstrated modest discrimination for all post-CPB hemodynamic variables, with the highest AUROC values observed for SI (0.723) and MSI (0.722), followed closely by SBP (0.717) and MBP (0.716); HR (0.643) and DBP (0.618) showed lower discrimination (Figure 3). The ROC-derived optimal cutoffs were 79.0 beats/min for HR, 99.6 mmHg for SBP, 73.4 mmHg for MBP, 64.0 mmHg for DBP, 0.755 for SI, and 1.208 for MSI. For clinical interpretability, these values were rounded to 80 beats/min, 100 mmHg, 70 mmHg, 60 mmHg, 0.7, and 1.2, respectively, for subsequent survival analyses.

Kaplan–Meier analyses (Figure S1) demonstrated significantly lower 30-day survival at the predefined thresholds (HR ≥ 80 beats/min, SBP < 100 mmHg, MBP < 70 mmHg, SI ≥ 0.7, and MSI ≥ 1.2; all *p* ≤ 0.013), whereas DBP less than 60 mmHg did not significantly stratify survival (*p* = 0.339). In restricted cubic spline analyses adjusted for Surgery group, CPB time and Mean VIS, mortality risk increased progressively above approximately 80 beats/min for HR and above 0.7 and 1.2 for SI and MSI, respectively, whereas risk increased below approximately 100 mmHg for SBP and below approximately 70 mmHg for MBP (Figure S2).

### 3.5. Post-CPB Hypotension Burden

Post-CPB hypotension burden below MBP 65 mmHg was consistently greater in non-survivors than in survivors (Table 4). The median AUC was 192.95 [84.04, 526.86] mmHg·min in non-survivors compared with 14.48 [0.00, 81.59] mmHg·min in survivors (*p* < 0.001). Similarly, TWA burden was higher in non-survivors (1.37 [0.55, 2.73] vs. 0.11 [0.00, 0.63] mmHg; *p* < 0.001), as was the percentage of time with MBP < 65 mmHg (22.20 [8.78, 33.43]% vs. 4.23 [0.00, 13.00]%; *p* < 0.001). These findings remained consistent in the sensitivity analysis restricted to patients with ≥80% MBP coverage.

## 4. Discussion

In this single-center cohort of adults undergoing aortic surgery with CPB, the most consistent between-group differences in routinely available hemodynamic variables were observed during the post-CPB phase. After Holm correction, significant between-group differences remained only for post-CPB SBP, MBP, SI, and MSI. In adjusted analyses, lower SBP and MBP, and higher SI and MSI were all independently associated with 30-day mortality. Specifically, each 10 mmHg decrease in SBP and MBP was associated with 60% and 100% higher mortality hazards, respectively; and each 0.1-unit increase in SI and MSI was associated with 38% and 29% higher mortality hazards, respectively. SBP, MBP, SI, and MSI showed similar discriminatory performance. Together, these findings suggest that the immediate post-CPB phase may represent a particularly informative window for hemodynamic risk stratification before transfer from the operating room.

This phase specificity is biologically plausible. Hemodynamics before incision and before CPB are influenced by transient factors such as anesthetic induction, airway manipulation, surgical stimulation, and ongoing titration of vasoactive medications [9,10,12,13]. By contrast, the post-CPB phase reflects the integrated physiological consequences of the operation itself, including myocardial ischemia–reperfusion, myocardial stunning, vasoplegia, hemodilution, bleeding, transfusion, and systemic inflammatory activation related to CPB [8,9,25,26,27]. Hemodynamic values during this phase may therefore reflect both the residual cardiovascular dysfunction after surgery and the effects of ongoing hemodynamic treatment.

Among the individual pressure variables, both SBP and MBP were informative. While their overall discriminatory power was comparable, spline analysis suggested a somewhat more pronounced increase in mortality risk below approximately 100 mmHg for SBP, whereas the increase below 70 mmHg for MBP was more gradual. One possible explanation is that systolic pressure after CPB more directly reflects the recovering ventricle’s ability to generate effective forward flow under conditions of altered vascular tone and pharmacologic support. Nevertheless, MBP remained associated with mortality and continues to be a familiar target for perfusion management, suggesting that both variables are clinically relevant.

HR showed an unadjusted association with mortality and stratified survival at the exploratory threshold of 80 beats/min; however, it was not independently associated with mortality in the adjusted model. Previous studies have evaluated HR in relation to adverse outcomes in perioperative and cardiovascular populations, although the populations, measurement windows, and outcomes differed [15,19,28,29,30,31,32,33,34,35,36]. At the same time, HR alone is intrinsically nonspecific after CPB. In this context, tachycardia may reflect compensation for reduced stroke volume, vasodilatory physiology, ongoing bleeding, inadequate preload, myocardial dysfunction, or increased catecholamine requirements. SI and MSI remained associated with mortality after adjustment, but the mechanisms underlying their associations were not directly evaluated in this study.

Prior studies differed from the present study in patient populations, measurement windows, and outcomes (Table 5). Reich et al. examined post-CPB hemodynamic extremes in CABG patients [19], while Jinadasa et al. evaluated blood pressure variability rather than phase-specific mean values [16]. Studies of discharge or outpatient HR focused on long-term outcomes among patients who survived the initial hospitalization or early postoperative period [28,31,34]. The reported associations were also not uniform: Ohnuma et al. found no significant association between early postoperative HR groups and long-term outcomes [33], and the fully adjusted mortality association in the study by Frank et al. was not significant [34]. These differences limit direct comparisons of thresholds and effect estimates with the present study of intraoperative post-CPB hemodynamics and 30-day mortality.

In contrast, DBP was less robust than the other measures. Although post-CPB DBP was lower in non-survivors and showed an unadjusted association with mortality, this relationship did not persist after adjustment. Additionally, the threshold of 60 mmHg did not significantly stratify survival. In the immediate post-CPB phase, diastolic pressure may be influenced more strongly by vasomotor tone, arterial compliance, and pulse pressure characteristics than by overall circulatory adequacy. This may limit its usefulness as a practical prognostic marker compared with SBP or composite indices.

These findings extend prior research in two ways. First, in complex aortic surgery, the prognostic value of routine hemodynamic variables appears to be highly dependent on timing. Early measurements are influenced by transient procedural effects, whereas the post-CPB phase reflects the combined effects of baseline cardiovascular reserve, operative injury, and ongoing hemodynamic treatment during recovery from CPB. Second, simple indices derived from universally available bedside variables performed at least as well as, and in some cases slightly better than, individual blood pressure measurements. From a clinical perspective, thresholds such as HR of 80 beats/min or greater, SBP less than 100 mmHg, MBP less than 70 mmHg, SI of 0.7 or greater, and MSI of 1.2 or greater may serve as pragmatic markers of increased risk before the patient leaves the operating room. These thresholds should not be interpreted as treatment targets. This observational study does not establish that correcting a hemodynamic abnormality beyond these thresholds will improve outcomes.

The medication data warrant cautious interpretation. Non-survivors more frequently received dobutamine before CPB and dobutamine, epinephrine, and vasopressin after CPB, suggesting greater hemodynamic instability and more intensive treatment in patients who ultimately died. These patterns most likely reflect confounding by indication rather than a direct drug effect. More broadly, the prognostic value of post-CPB HR and blood pressure may lie in what these abnormalities indicate about the severity of circulatory dysfunction and level of the support required to maintain separation from CPB. Although mean post-CPB VIS was included in the adjusted models, residual confounding related to treatment indication and other aspects of intraoperative management may remain. Hemodynamic values and vasoactive support were assessed during the same phase, limiting the separation of underlying circulatory dysfunction from the effects of ongoing treatment.

The additional hypotension-burden analysis further demonstrated that non-survivors experienced not only lower average post-CPB MBP but also a greater cumulative depth and duration of hypotension below 65 mmHg. These findings describe the greater temporal burden of post-CPB hypotension in non-survivors; however, its incremental prognostic value beyond mean blood pressure was not evaluated.

Several limitations should be acknowledged. This was a retrospective single-center study, which limits causal inference and generalizability. Exclusion of patients with incomplete intraoperative recordings may have introduced selection bias if recording completeness was related to clinical severity or outcomes. The cohort included heterogeneous aortic procedures and indications, and only 30 deaths occurred within 30 days, limiting precision despite ridge penalization and multivariable adjustment. Residual confounding from baseline patient characteristics and other aspects of perioperative management, including fluid balance and transfusion, may remain despite adjustment for surgery group, CPB time, and mean VIS. The pre-incision phase included anesthesia induction, tracheal intubation, and subsequent stabilization, and averaging measurements across this heterogeneous interval may have attenuated transient hemodynamic changes and their associations with mortality. Hemodynamic variables were summarized as phase-wise means from 5 min interval recordings. Although post-CPB hypotension burden was additionally estimated using linear interpolation, brief hemodynamic changes may not have been fully captured, and the estimates may have been affected by missing intervals. We also did not evaluate postoperative hemodynamic trajectories or biochemical markers of hypoperfusion, which may have provided additional mechanistic insight. Finally, the proposed thresholds were internally derived and should be considered hypothesis-generating until externally validated.

## 5. Conclusions

In summary, routinely available hemodynamic variables measured after CPB, particularly SBP, MBP, SI, and MSI, were associated with 30-day mortality following aortic surgery. These findings highlight the immediate post-CPB phase as a practical window for risk stratification before leaving operating-room and warrant external validation.

## Figures and Tables

**Table 1:** Baseline characteristics by all-cause 30-day mortality.

Variables	All Patients (*n* = 691)	Survival (*n* = 661)	Non-Survival (*n* = 30)	SMD	*p*-Value
**Demographic**					
Age; years	64.0 [53.0, 73.0]	64.0 [52.0, 72.0]	75.0 [67.2, 77.0]	0.949	<0.001
Sex; male	485 (70.2)	467 (70.7)	18 (60.0)	0.225	0.297
Height; cm	165.9 [159.3, 172.9]	166.4 [159.6, 173.0]	159.9 [154.0, 164.8]	0.716	<0.001
Weight; kg	67.1 [58.6, 75.8]	67.4 [58.9, 76.0]	60.9 [56.9, 66.0]	0.628	0.003
BMI; kg/m^2^	24.4 [22.3, 26.5]	24.4 [22.2, 26.5]	23.8 [22.5, 25.6]	0.177	0.384
ASA > III	118 (17.1)	106 (16.0)	12 (40.0)	0.554	0.002
Emergency	92 (13.3)	81 (12.3)	11 (36.7)	0.592	0.001
**Surgical category**				0.269	0.224
Root/ascending-only surgery	293 (42.4)	284 (43.0)	9 (30.0)		
Arch and/or distal aortic surgery	398 (57.6)	377 (57.0)	21 (70.0)		
**Comorbidity**					
DM	68 (9.8)	64 (9.7)	4 (13.3)	0.115	0.525
Hypertension	324 (46.9)	313 (47.4)	11 (36.7)	0.218	0.337
**Duration of operation**					
Procedure time	390.0 [317.5, 512.5]	385.0 [316.0, 500.0]	592.5 [447.8, 670.0]	1.072	<0.001
CPB time	148.0 [109.0, 210.0]	145.0 [108.0, 207.0]	244.0 [181.5, 296.8]	0.982	<0.001
**Post-CPB**					
Mean VIS	4.0 [0.4, 9.4]	3.7 [0.3, 8.9]	16.29 [4.9, 29.5]	0.572	<0.001
Max VIS	5.0 [2.0, 15.0]	5.0 [2.0, 13.0]	28.50 [9.3, 45.0]	0.272	<0.001

Abbreviations: Standardized Mean Difference; SMD, BMI, body mass index calculated as weight in kilograms divided by height in meters squared; ASA, American Society of Anesthesiologists. VIS, vasoactive-inotropic score. Data are presented as the median [Q1, Q3], the mean ± Standard deviation and number (%) as appropriate. Procedure time and CPB time unit are min. Surgical category was defined according to the anatomical extent of aortic surgery. Procedures limited to the aortic root and/or ascending aorta were classified as root/ascending-only surgery. Procedures involving the aortic arch and/or distal aorta, with or without concomitant root or ascending aortic procedures, were classified as arch and/or distal aortic surgery. Mean and maximum VIS were calculated during the post-CPB phase.

**Table 2:** Perioperative hemodynamic variables according to 30-day mortality.

Variables	All Patients (*n* = 691)	Survival (*n* = 661)	Non-Survival (*n* = 30)	*p*-Value	Holm-Adjusted *p*-Value
**Preoperative**					
Heart rate	69.7 [63.7, 76.3]	69.6 [63.7, 76.1]	71.0 [63.7, 82.5]	0.396	1.000
SBP	122.7 [112.7, 133.7]	122.6 [112.7, 133.7]	123.4 [114.2, 128.6]	0.907	1.000
MBP	87.3 [81.0, 94.5]	87.3 [81.0, 94.5]	88.6 [81.0, 94.7]	0.996	1.000
DBP	69.9 ± 9.9	69.9 ± 9.9	69.0 ± 9.5	0.586	1.000
SI	0.6 [0.5, 0.6]	0.6 [0.5, 0.6]	0.6 [0.5, 0.6]	0.927	1.000
MSI	0.8 [0.7, 0.9]	0.8 [0.7, 0.9]	0.8 [0.7, 0.9]	0.597	1.000
**Intraoperative**					
Pre-incision					
Heart rate	65.6 [59.2, 74.1]	65.6 [59.2, 73.6]	73.7 [61.2, 82.7]	0.067	0.904
SBP	108.9 [101.3, 117.6]	109.1 [101.4, 118.1]	105.2 [98.4, 110.1]	0.063	0.904
MBP	74.8 [68.8, 81.3]	74.8 [68.8, 81.6]	73.0 [67.6, 75.9]	0.075	0.904
DBP	57.5 [50.3, 63.4]	57.6 [50.3, 63.8]	56.2 [48.6, 59.8]	0.203	1.000
SI	0.6 [0.5, 0.7]	0.6 [0.5, 0.7]	0.6 [0.5, 0.8]	0.056	0.904
MSI	0.9 [0.8, 1.0]	0.9 [0.8, 1.0]	1.0 [0.8, 1.2]	0.026	0.487
Pre-CPB					
Heart rate	66.8 [59.7, 76.0]	66.5 [59.6, 75.6]	73.7 [63.9, 86.4]	0.027	0.495
SBP	100.8 [91.5, 108.7]	100.7 [91.5, 108.7]	104.4 [91.6, 109.3]	0.348	1.000
MBP	72.2 [65.7, 77.9]	72.2 [65.8, 77.8]	73.3 [63.3, 79.6]	0.908	1.000
DBP	57.1 ± 9.2	57.2 ± 9.2	55.1 ± 9.4	0.250	1.000
SI	0.7 [0.6, 0.8]	0.7 [0.6, 0.8]	0.7 [0.6, 0.9]	0.242	1.000
MSI	0.9 [0.8, 1.1]	0.9 [0.8, 1.1]	1.0 [0.8, 1.3]	0.058	0.904
Post-CPB					
Heart rate	78.9 [70.5, 87.4]	78.5 [70.2, 87.3]	84.3 [79.2, 97.9]	0.008	0.164
SBP	110.8 [103.4, 117.4]	110.9 [103.9, 117.9]	98.9 [95.1, 111.0]	<0.001	0.001
MBP	79.4 [74.2, 85.2]	79.7 [74.7, 85.4]	72.7 [69.2, 80.1]	<0.001	0.001
DBP	63.1 [57.6, 68.4]	63.3 [57.7, 68.5]	60.6 [52.4, 63.9]	0.028	0.495
SI	0.7 [0.6, 0.8]	0.7 [0.6, 0.8]	0.8 [0.7, 1.1]	<0.001	<0.001
MSI	1.0 [0.9, 1.1]	1.0 [0.9, 1.1]	1.2 [1.0, 1.4]	<0.001	<0.001

Abbreviations: SBP, systolic blood pressure; MBP, mean blood pressure; DBP, diastolic blood pressure; CPB, cardiopulmonary bypass; SI, shock index; MSI, modified shock index. Data are presented as the median [Q1, Q3] or the mean ± Standard deviation as appropriate. Heart rate is expressed in beats/min and blood pressure in mmHg. Holm-adjusted *p*-values were calculated across the 24 between-group comparisons of six hemodynamic variables across four perioperative phases.

**Table 3:** Univariable and multivariable Cox proportional hazards models of post-CPB hemodynamic variables for 30-day mortality.

	Univariable		Multivariable	
	Crude Hazard Ratio (95% CI)	*p*-Value	Adjusted Hazard Ratio (95% CI)	*p*-Value
Heart Rate; 10 bpm	1.42 (1.13, 1.80)	0.003	1.24 (0.98, 1.56)	0.070
SBP; −10 mmHg	1.96 (1.52, 2.54)	<0.001	1.60 (1.24, 2.05)	<0.001
MBP; −10 mmHg	2.61 (1.66, 4.10)	<0.001	2.00 (1.32, 3.03)	0.001
DBP; −10 mmHg	1.67 (1.06, 2.64)	0.026	1.45 (0.96, 2.21)	0.080
SI; 0.1 unit	1.59 (1.35, 1.89)	<0.001	1.38 (1.17, 1.64)	<0.001
MSI; 0.1 unit	1.45 (1.27, 1.67)	<0.001	1.29 (1.12, 1.47)	<0.001

Each model included one post-CPB hemodynamic variable. Multivariable models were adjusted for surgery group, CPB time, and post-CPB Mean VIS and were estimated using ridge-penalized Cox regression with an L2 penalty (penalizer = 0.01). HR was modeled per 10 beats/min increase, SBP, MBP, and DBP per 10 mmHg decrease, and SI and MSI per 0.1 unit increase.

**Table 4:** Post-CPB MBP < 65 mmHg hypotension burden according to 30-day mortality.

Variables	All Patients (*n* = 691)	Survival (*n* = 661)	Non-Survival (*n* = 30)	*p*-Value
**Post-CPB MBP < 65 mmHg**				
AUC, mmHg·min	17.68 [0.00, 96.48]	14.48 [0.00, 81.59]	192.95 [84.04, 526.86]	<0.001
TWA, mmHg	0.13 [0.00, 0.70]	0.11 [0.00, 0.63]	1.37 [0.55, 2.73]	<0.001
Time below threshold, %	4.80 [0.00, 14.05]	4.23 [0.00, 13.00]	22.20 [8.78, 33.43]	<0.001

Abbreviations: MBP, mean blood pressure; AUC, area under the MBP threshold; TWA, time-weighted average. Data is presented as the median [Q1, Q3]. AUC represents the integrated deficit of MBP below 65 mmHg during the post-CPB phase. The reported *p*-values represent unadjusted comparisons between survivors and non-survivors.

**Table 5:** Selected studies of perioperative hemodynamics and outcomes after cardiac or aortic surgery.

Study and Population	Measurement Window and Exposure	Outcomes and Key Estimates	Comparison with the Present Study
Reich et al. (1999) [19] 2149 primary CABG patients; two centers. Mortality model: *n* = 2066.	Pre-, intra-, and post-CPB periods. Proportion of 2 min epochs within hemodynamic extremes; only MAP analyzed during CPB. Very high post-CPB HR: >120 beats/min.	In-hospital mortality, stroke, and perioperative myocardial infarction. Post-CPB very high HR: reported mortality aOR 3.1 (1.5–6.1), *p* = 0.001. The modeled exposure contrast is not specified ^a^.	Provides prior evidence for post-CPB assessment. Examined CABG and exposure to hemodynamic extremes; the present study examines aortic surgery, phase means, and SI/MSI.
Jinadasa et al. (2018) [16] 3687 adults undergoing cardiac surgery with CPB; single center.	CV-SBP: pre- and post-CPB, separately and combined; active CPB excluded. CV-MAP: all three phases and the whole case. CV = SD/mean; modeled per 0.10 increase.	30-day mortality and in-hospital renal failure. Combined pre/post CV-SBP: mortality aOR 2.50 (1.60–3.92); renal failure 2.04 (1.33–3.14). Post-CPB CV-SBP: mortality 1.40 (0.97–2.04), *p* = 0.08; renal failure 1.56 (1.09–2.22), *p* = 0.01 ^f^.	Assessed BP variability. Post-CPB CV-SBP was associated with renal failure but not significantly with mortality. The present study evaluates phase means, HR, and SI/MSI.
Frank et al. (2010) [34] 794 patients after nonurgent isolated CABG; first-month deaths excluded. Included off-pump CABG.	Resting ECG-derived HR at the first outpatient visit, scheduled 2–3 months after surgery. Highest HR category ≥ 90 vs. <60 beats/min ^b^.	All-cause mortality and a composite of death or cardiovascular events; mean follow-up 3.2 years. Fully adjusted Model 4: Mortality aHR 3.57 (0.90–14.17), *p* = 0.07. Composite aHR 2.26 (1.04–4.91), *p* = 0.04.	Assessed outpatient HR and long-term events among early survivors. The fully adjusted mortality association was not significant. It does not validate an intraoperative 30-day mortality cutoff.
Ohnuma et al. (2015) [33] 434 patients after acute type A dissection repair.	Mean HR from readings at 06:00, 14:00, and 20:00 on postoperative days 1, 3, 5, and 7. Groups < 70, 70–79, 80–89, and >90 beats/min, as printed ^h^.	10-year survival and aortic events; median follow-up 52 months. No statistically significant association with HR group was reported. Adjusted HR-category estimates/CIs and the complete Cox adjustment set were not reported.	Assessed several postoperative days and long-term outcomes. Early deaths were excluded. Nonsignificant findings do not establish equivalence or exclude association with early mortality.
Hagiya et al. (2021) [31] 721 hospital survivors after acute type A dissection repair; single center.	Last recorded HR within 24 h before discharge. Quartiles < 70, 70–77, 78–83, and ≥84 beats/min.	Late aortic events; median observation 5.8 years. HR ≥ 84 vs. <70 beats/min: Model 4 aHR 1.86 (1.06–3.25), *p* = 0.029. Overall survival did not differ significantly across groups, log-rank *p* = 0.905 ^c^.	Assessed discharge HR among hospital survivors. The positive association concerned a long-term aortic composite, rather than 30-day all-cause mortality.
Ho et al. (2025) [28] 839 hospital survivors after acute type A dissection repair; 597 analyzed at 3 months.	HR, SBP, and DBP at discharge and at the 3-month visit. HR ≤ 80, 81–100, >100 beats/min; SBP < 130, 130–150, >150 mmHg.	Long-term all-cause mortality, aortic progression, and aortic surgery; median follow-up 2.8 years. Mortality aHRs for discharge measurements: SBP > 150 vs. <130, 3.21 (1.61–6.37); HR 81–100 vs. ≤80, 2.59 (1.28–5.24). For 3-month measurements: HR > 100 vs. ≤80, 3.59 (1.19–10.78) ^g^.	Assessed discharge and outpatient vital signs in hospital survivors. Three months denotes exposure assessment, not the mortality endpoint. The present study examines an earlier intraoperative window.
Present study 691 adults undergoing aortic surgery with CPB; single center	Preoperative, pre-incision, pre-CPB, and post-CPB phase means; primary analyses focused on the post-CPB window, from CPB termination to anesthesia end. Intraoperative HR/BP recorded every 5 min; SI and MSI calculated from phase-specific mean HR and BP. Active CPB excluded. Additional post-CPB MBP < 65 mmHg burden measures: AUC, TWA burden, and percentage of time below threshold. Exploratory thresholds used: HR ≥ 80 beats/min; SBP < 100, MBP < 70, DBP < 60 mmHg; SI ≥ 0.7; MSI ≥ 1.2 ^d^.	30-day all-cause mortality: 30/691 (4.3%). Separate ridge-penalized Cox models, aHR (95% CI) ^e^: HR, per 10 beats/min higher: 1.24 (0.98–1.56), *p* = 0.070. SBP, per 10 mmHg lower: 1.60 (1.24–2.05), *p* < 0.001. MBP, per 10 mmHg lower: 2.00 (1.32–3.03), *p* = 0.001. DBP, per 10 mmHg lower: 1.45 (0.96–2.21), *p* = 0.080. SI, per 0.1-unit higher: 1.38 (1.17–1.64), *p* < 0.001. MSI, per 0.1-unit higher: 1.29 (1.12–1.47), *p* < 0.001. Post-CPB hypotension burden was greater in non-survivors for all three measures (all unadjusted between-group *p* < 0.001).	Evaluates phase-specific hemodynamics, HR-derived indices, and post-CPB hypotension burden in an aortic surgery cohort, with a 30-day all-cause mortality endpoint.

Abbreviations: aHR, adjusted hazard ratio; aOR, adjusted odds ratio; AUC, area under the MBP threshold; BP, blood pressure; CABG, coronary artery bypass grafting; CI, confidence interval; CPB, cardiopulmonary bypass; CV, coefficient of variation; DBP, diastolic blood pressure; ECG, electrocardiogram; HR, heart rate; MAP, mean arterial pressure; MBP, mean blood pressure; MSI, modified shock index (HR/MBP); ROC, receiver-operating characteristic; SBP, systolic blood pressure; SD, standard deviation; SI, shock index (HR/SBP); TWA, time-weighted average; VIS, vasoactive-inotropic score. ^a^ Reich et al.: very high HR was >120 beats/min (Table 1). Table 4 reports aOR 3.1 (1.5–6.1), but does not specify the increment/reference for that HR term. Mortality was during the index hospitalization; 50 deaths occurred in the full cohort and 48 in the mortality model. ^b^ Frank et al.: Table 2 and Table 3 label the highest category ≥ 90 beats/min, whereas the abstract/text use > 90. Both estimates are from fully adjusted Model 4. The composite comprises death, nonfatal acute coronary syndromes, nonfatal stroke/transient ischemic attack, repeat coronary revascularization, and vascular surgery. ^c^ Hagiya et al.: late aortic events include aneurysm-related or sudden death, aortic surgery, and hospitalization for recurrent acute dissection. The ≥84 beats/min quartile contrast is distinct from the ROC-derived cutoff of 81 beats/min. The mortality comparison quoted here is an unadjusted log-rank test. ^d^ Present-study thresholds were used for exploratory Kaplan–Meier analyses. Exact ROC-derived cutoffs were HR 79.0 beats/min, SBP 99.6 mmHg, MBP 73.4 mmHg, DBP 64.0 mmHg, SI 0.755, and MSI 1.208. DBP < 60 mmHg did not significantly stratify survival (log-rank *p* = 0.339). These internally derived thresholds require external validation and should not be interpreted as treatment targets. ^e^ Each present-study model included one hemodynamic variable and was adjusted for surgery group, CPB duration, and mean VIS using ridge-penalized Cox regression (L2 penalty; penalizer = 0.01). Estimates are adjusted hazard ratios with 95% confidence intervals. ^f^ Jinadasa et al.: pre-CPB CV-SBP was associated with mortality (aOR 1.75 [1.17–2.62], *p* = 0.01). Adjusted CV-MAP estimates were not significant for either outcome. Primary analyses used *p* < 0.0125; phase analyses were exploratory and used *p* < 0.05. Eligibility included nonvalvular aortic procedures, so this was a mixed cardiac cohort rather than an explicitly non-aortic cohort. ^g^ Ho et al.: discharge HR > 100 vs. ≤80 beats/min was not significantly associated with mortality (aHR 2.09 [0.77–5.67]), although it was associated with later aortic surgery (2.19 [1.24–3.86]). Do not interpret the discharge HR result as a proven monotonic mortality gradient. DBP boundary labels differ between Methods and Table 2 and Table 3. ^h^ Ohnuma et al.: aortic events included rupture, secondary operation, endovascular stenting, late organ ischemia and sudden death. The highest HR group is printed as >90 beats/min; assignment of exactly 90 is not explained.

## Data Availability

The data that support the findings of this study are not publicly available because they contain potentially identifiable patient information and are subject to institutional restrictions on public sharing, but may be available from the corresponding author upon reasonable request and with institutional approval. The study-specific analysis code is also subject to institutional restrictions on public release and is therefore not publicly available.

## Supplementary Materials

The following supporting information can be downloaded at: https://www.mdpi.com/article/10.3390/jcm15207908/s1. Table S1: Intraoperative medication uses according to 30-day mortality; Table S2: Univariable and Ridge-penalized multivariable Cox proportional hazards models of post-CPB hemodynamic variables for 30-day mortality; Figure S1: Kaplan–Meier analyses of all-cause 30-day mortality according to clinically rounded post-CPB hemodynamic thresholds; Figure S2: Adjusted restricted cubic spline Cox models for associations between post-CPB hemodynamic variables and all-cause 30-day mortality.
