# Supplementary material for: Association Between Post-Cardiopulmonary Bypass Hemodynamics and 30-Day Mortality After Aortic Surgery: A Single-Center, Retrospective Study
Source: J Clin Med. 2026 Oct 13;15(20):7908. doi: 10.3390/jcm15207908 (PMC13706227; doi:10.3390/jcm15207908)
Supplement: Supplementary file 1 [file jcm-15-07908-s001.zip › jcm-4541928-supplementary.pdf]

Supplemental Digital Content

Table S1. Intraoperative medication uses according to 30-day mortality.

**Variables**	**All patients (n = 691)**	**Survival (n = 661)**	**Non-survival (n = 30)**	***P*-value**
**Intraoperative**				
Pre-incision				
Vasopressors	251 (36.3)	236 (35.7)	15 (50)	0.162
Dobutamine	17 (2.5)	14 (2.1)	3 (10)	0.033
Dopamine	1 (0.1)	1 (0.2)	0 (0)	1.000
Ephedrine	2 (0.3)	1 (0.2)	1 (3)	0.085
Epinephrine	5 (0.7)	5 (0.8)	0 (0)	1.000
Isoproterenol	1 (0.1)	1 (0.2)	0 (0)	1.000
Milrinone	1 (0.1)	1 (0.2)	0 (0)	1.000
Norepinephrine	248 (35.9)	232 (35.1)	16 (53)	0.065
Phenylephrine	2 (0.3)	2 (0.3)	0 (0)	1.000
Vasopressin	2 (0.3)	2 (0.3)	0 (0)	1.000
Antihypertensives	7(1.0)	5 (0.8)	2 (7)	0.033
Pre-CPB				
Vasopressors	319 (46.2)	301 (45.5)	18 (60)	0.172
Dobutamine	22 (3.2)	18 (2.7)	4 (13)	0.012
Dopamine	1 (0.1)	1 (0.2)	0 (0)	1.000
Ephedrine	0 (0)	0 (0)	0 (0)	1.000
Epinephrine	6 (0.9)	5 (0.8)	1 (3)	0.235
Isoproterenol	1 (0.1)	1 (0.2)	0 (0)	1.000
Milrinone	0 (0)	0 (0)	0 (0)	1.000
Norepinephrine	316 (45.7)	297 (44.9)	19 (63)	0.073

Phenylephrine	1 (0.1)	1 (0.2)	0 (0)	1.000
Vasopressin	5 (0.7)	4 (0.6)	1 (3)	0.200
Antihypertensives	4 (0.6)	4 (0.6)	0 (0)	1.000
Post-CPB				
Vasopressors	630 (91.2)	602 (91.1)	28 (93)	1.000
Dobutamine	22 (3.2)	18 (2.7)	4 (13)	0.008
Dopamine	0 (0)	0 (0)	0 (0)	1.000
Ephedrine	2 (0.3)	2 (0.3)	0 (0)	1.000
Epinephrine	108 (15.6)	92 (13.9)	16 (53)	< 0.001
Isoproterenol	0 (0)	0 (0)	0 (0)	1.000
Milrinone	8 (1.2)	8 (1.2)	0 (0)	1.000
Norepinephrine	620 (89.7)	593 (89.7)	27 (90)	1.000
Phenylephrine	1 (0.1)	1 (0.2)	0 (0)	1.000
Vasopressin	35 (5.1)	26 (3.9)	9 (30)	< 0.001
Antihypertensives	3 (0.4)	3 (0.5)	0 (0)	1.000

*Data are presented as N (%).

Table S2. Univariable and Ridge-penalized multivariable Cox proportional hazards models of post-CPB hemodynamic variables for 30-day mortality.

	**Univariable**		**Multivariable**	
	**Crude Hazard Ratio (95% CI)**	***P*-value**	**Adjusted Hazard Ratio (95% CI)**	***P*-value**
Surgery group	1.76 (0.81, 3.85)	0.155	2.01 (1.01, 4.01)	0.047
CPB time; 10 mins	1.08 (1.05, 1.11)	< 0.001	1.07 (1.04, 1.09)	< 0.001
Mean VIS; 10 unit	1.07 (1.01, 1.12)	0.017	1.07 (1.00, 1.15)	0.037
Heart Rate; 10 bpm	1.42 (1.13, 1.80)	0.003	1.24 (0.98, 1.56)	0.070
Surgery group	1.76 (0.81, 3.85)	0.155	2.13 (1.07, 4.23)	0.032
CPB time; 10 mins	1.08 (1.05, 1.11)	< 0.001	1.06 (1.03, 1.09)	< 0.001
Mean VIS; 10 unit	1.07 (1.01, 1.12)	0.017	1.07 (1.00, 1.16)	0.065
SBP; -10 mmHg	1.96 (1.52, 2.54)	< 0.001	1.60 (1.24, 2.05)	< 0.001
Surgery group	1.76 (0.81, 3.85)	0.155	2.05 (1.03, 4.09)	0.041
CPB time; 10 mins	1.08 (1.05, 1.11)	< 0.001	1.07 (1.04, 1.10)	< 0.001
Mean VIS; 10 unit	1.07 (1.01, 1.12)	0.017	1.07 (1.00, 1.15)	0.057
MBP; -10 mmHg	2.61 (1.66, 4.10)	< 0.001	2.00 (1.32, 3.03)	0.001
Surgery group	1.76 (0.81, 3.85)	0.155	1.92 (0.96, 3.84)	0.067
CPB time; 10 mins	1.08 (1.05, 1.11)	< 0.001	1.07 (1.05, 1.10)	< 0.001

Mean VIS; 10 unit	1.07 (1.01, 1.12)	0.017	1.07 (1.00, 1.15)	0.037
DBP; -10 mmHg	1.67 (1.06, 2.64)	0.026	1.45 (0.96, 2.21)	0.080
Surgery group	1.76 (0.81, 3.85)	0.155	2.03 (1.02, 4.04)	0.043
CPB time; 10 mins	1.08 (1.05, 1.11)	< 0.001	1.06 (1.03, 1.09)	< 0.001
Mean VIS; 10 unit	1.07 (1.01, 1.12)	0.017	1.07 (1.00, 1.15)	0.061
SI; 0.1 unit	1.59 (1.35, 1.89)	< 0.001	1.38 (1.17, 1.64)	< 0.001
Surgery group	1.76 (0.81, 3.85)	0.155	1.95 (0.98, 3.89)	0.056
CPB time; 10 mins	1.08 (1.05, 1.11)	< 0.001	1.06 (1.03, 1.09)	< 0.001
Mean VIS; 10 unit	1.07 (1.01, 1.12)	0.017	1.07 (1.00, 1.15)	0.060
MSI; 0.1 unit	1.45 (1.27, 1.67)	< 0.001	1.29 (1.12, 1.47)	< 0.001

Abbreviations: CPB, cardiopulmonary bypass; Mena VIS, mean vasoactive-inotropic score; SBP, systolic blood pressure; MBP, mean blood pressure; SI, shock index; MSI, modified shock index.

*Multivariable models were adjusted for surgery group, CPB time, and mean VIS and were estimated using Ridge-penalized Cox regression (L2 penalty, penalizer = 0.01). HR was modeled per 10-beats/min increase, SBP, MBP, and DBP per 10 mmHg decrease, and SI and MSI per 0.1-unit increase.

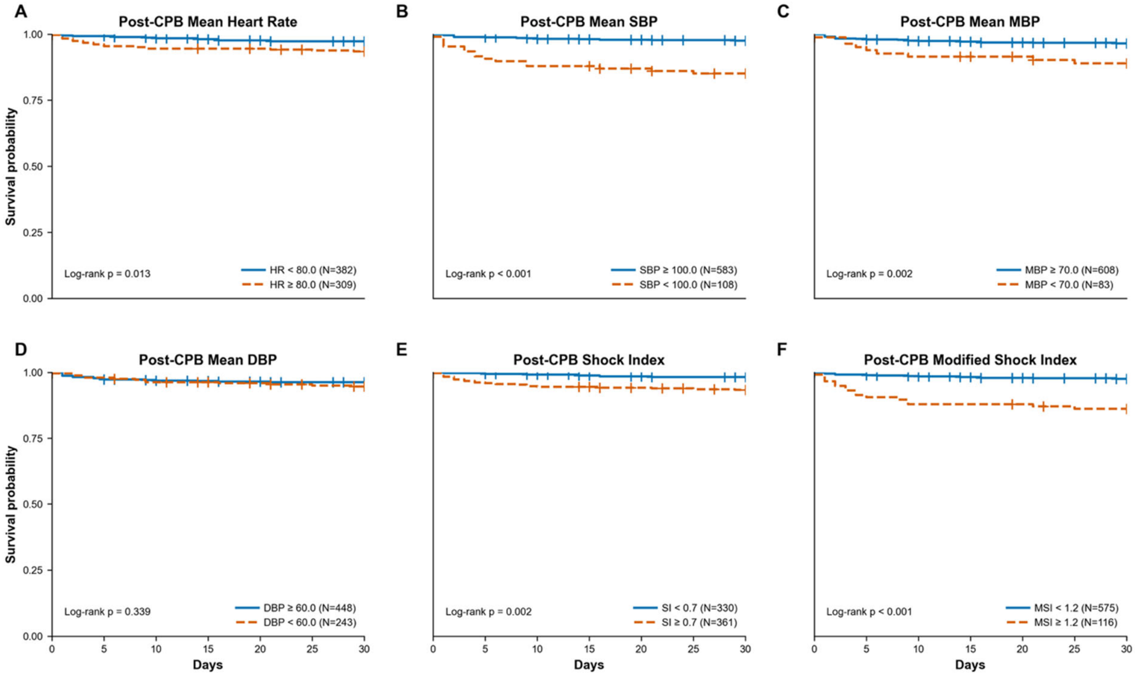

Figure S1. Kaplan–Meier analyses of all-cause 30-day mortality according to clinically rounded post-CPB hemodynamic thresholds.

Kaplan–Meier curves are shown for (A) heart rate (<80 vs. ≥80 beats/min), (B) systolic blood pressure (≥100 vs. <100 mmHg), (C) mean blood pressure (≥70 vs. <70 mmHg), (D) diastolic blood pressure (≥60 vs. <60 mmHg), (E) shock index (<0.7 vs. ≥0.7), and (F) modified shock index (<1.2 vs. ≥1.2). Lower 30-day survival was observed in patients with heart rate ≥80 beats/min (log-rank P = 0.013), systolic blood pressure <100 mmHg (P < 0.001), mean blood pressure <70 mmHg (P = 0.002), shock index ≥0.7 (P = 0.002), and modified shock index ≥1.2 (P < 0.001). Diastolic blood pressure <60 mmHg did not significantly stratify survival (P = 0.339). Tick marks indicate censoring.

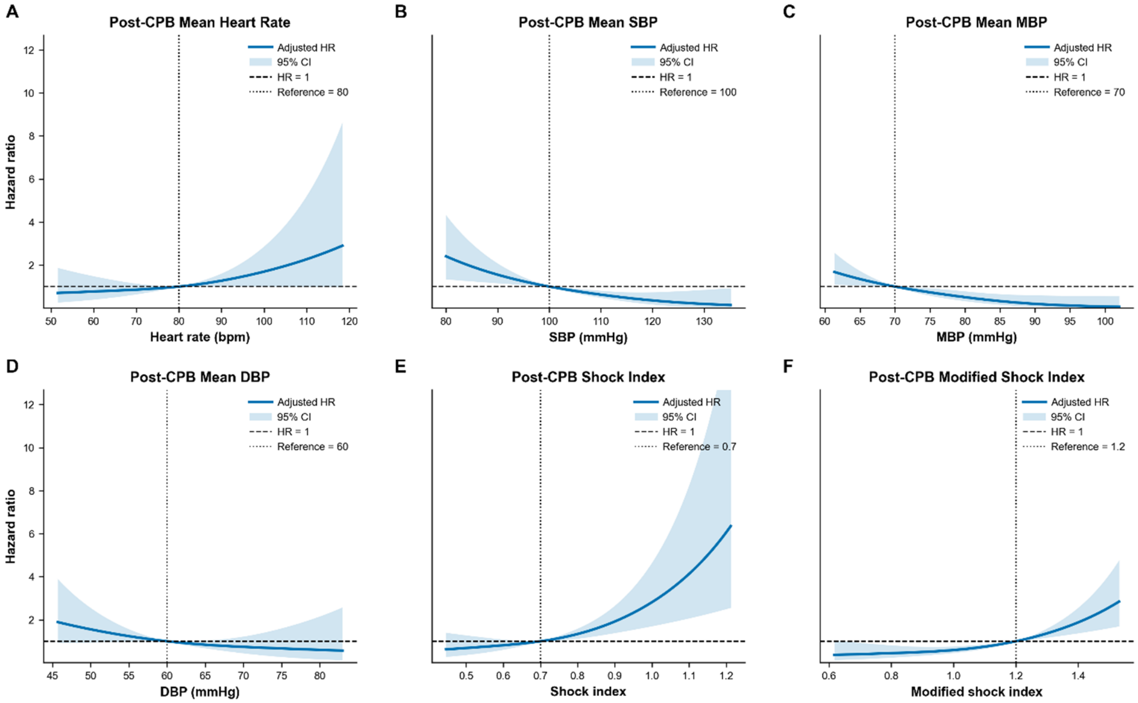

Figure S2. Adjusted restricted cubic spline Cox models for associations between post-CPB hemodynamic variables and all-cause 30-day mortality.

Restricted cubic spline Cox models are shown for post-CPB heart rate, systolic blood pressure, mean blood pressure, diastolic blood pressure, shock index, and modified shock index. Models were adjusted for surgery group, CPB time, and mean VIS. Solid lines indicate adjusted hazard ratios, shaded areas indicate 95% CIs, the horizontal dashed line indicates a hazard ratio of 1.0, and the vertical dotted line indicates the reference value for each variable. Abbreviations: CPB, cardiopulmonary bypass; Mean VIS, mean vasoactive-inotropic score; SBP, systolic blood pressure; MBP, mean blood pressure; SI, shock index; MSI, modified shock index; CI, confidence interval.
